# Effect of combined skin-to-skin contact, breastfeeding, and parents’ live lullaby singing on relieving acute procedural pain in neonates (SWEpap): a multicenter randomized controlled trial in Sweden

**DOI:** 10.1186/s12887-025-06393-y

**Published:** 2025-12-10

**Authors:** Martina Carlsen Misic, Jenny Ericson, Mats Eriksson, Emma Olsson, Alexandra Ullsten

**Affiliations:** 1 Department of Pediatrics, Faculty of Medicine and Health, Örebro University, Örebro, Sweden; 2 Faculty of Medicine and Health, School of Health Sciences, Örebro University, Örebro, Sweden; 3 Department of Health and Welfare, Dalarna University, Falun, Sweden; 4 Centre for Clinical Research and Education, Region Värmland, Karlstad, Sweden

**Keywords:** Breastfeeding, Galvanic skin response, Infant, Infant-directed live singing, Neonate, Pain management, Parent, Parent-delivered intervention, PIPP-R, Skin-to-skin contact

## Abstract

**Background:**

Engaging parents in parent-delivered pain relief in routine postnatal care is aligned with evidence-informed infant pain care and should be encouraged. This is part two of the mixed-methods SWEpap research project investigating combined parent-delivered pain management. Skin-to-skin contact (SSC) and breastfeeding are among the most studied parental pain-relieving interventions and are often combined for better effects. Live parental lullaby singing has not previously been investigated in combination with SSC and breastfeeding during painful procedures. This study investigated the efficacy of combined parent-delivered pain management versus standard care with oral glucose in healthy newborn infants during routine venipuncture.

**Methods:**

This was a multicenter randomized controlled trial with three parallel groups. Parent–infant dyads (*n*= 225) were recruited from three healthcare regions in Sweden and randomized to one of the following three groups during routine venipuncture: 1) standard care with glucose; 2) SSC with parent; and 3) combination of SSC, breastfeeding (if applicable), and parents’ live singing. The primary outcome was pain expression assessed using Premature Infant Pain Profile—Revised (PIPP-R); the secondary outcomes were galvanic skin response (GSR) and parents’ evaluations of pain, stress, and meaningfulness using a visual analogue scale (VAS).

**Results:**

The median PIPP-R was 5 (IQR 3–6) for group 1, 7 (5–9) for group 2, and 7 (5–10) for group 3 (*p*< 0.001). There were no significant differences in GSR. Parents’ VAS-assessment of infant pain ranged from a median of 9.5 to 17 mm, significantly higher in groups 2 and 3 (*p*= 0.017). The parents rated their own stress 4.5 - 6.5 (n.s.) and meaningfulness 93 - 96 (n.s.).

**Conclusion:**

Pain scores remained within the mild to moderate range across all groups, with the infants receiving oral glucose having significantly lower pain scores. This was the first study combining SSC, breastfeeding, and parents’ live lullaby singing, and more research is needed to optimize parent-delivered pain management. Parent-delivered pain management combining SSC, breastfeeding, and parents’ live lullaby singing is a feasible and safe intervention with potential pain alleviating properties offering the parents a strong sense of meaningfulness and stress relief.

**Trial registration:**

Clinical trial number: NCT04341194 Trial registration date: 10-04-2020 Trial start date: 01-03-2021

**Supplementary Information:**

The online version contains supplementary material available at 10.1186/s12887-025-06393-y.

## Background

During the first period of life, hospitalized infants as well as healthy newborns infants experience recurrent painful procedures. This study is the second part of the interdisciplinary multicenter SWEpap (Parents as pain management in Swedish neonatal care) project. The first part was a feasibility study exploring parents’ and nurses’ perceptions of and reflections on experiencing combined parent-delivered pain management in. hospitalized infants with skin-to-skin contact (SSC), breastfeeding (if applicable), and live parental lullaby singing [1]. The qualitative results of SWEpap part 1 [1] showed that mental and practical preparations are central to implementing combined parent-delivered pain management. Preparation, participation, and closeness were facilitated by the parents’ live lullaby singing, and both parents and nurses found the combined parent-delivered methods feasible for promoting self-efficacy and confidence in both parents and nurses [1]. Most parents express a desire and readiness to take an active role in protecting their infants from pain by helping them manage routine painful procedures [2]. Parents are a valuable but underutilized resource in procedural pain management for infants, and parents’ participation in pain management is currently being examined in neonatal pain research [3]. A recent global survey, investigating the inclusion of parent-delivered pain management in clinical practice on a day-to-day basis, reported that most of the responding neonatal units had local guidelines for neonatal pain management, but only a minority of these guidelines recommended parent-delivered pain management and assessment [4]. However, most of the responding units were positive toward involving parents in pain management and reported that parents regularly or always performed some form of pain management [4].

Several parent-delivered pain management methods are considered safe, cost-effective, and efficient for relieving procedural pain in sick and healthy infants. Many studies have demonstrated the pain-alleviating effect of SSC on both preterm and term infants [5]. Breastfeeding has also been shown to be effective at relieving neonatal pain with an effect comparable to or greater than that of sweet solutions administered during venipuncture in full-term infants [6]. A combination of several non-pharmacological pain management methods is considered more effective than individual parent-delivered interventions, indicating a presumed additive analgesic effect [7]. However, more research on the efficacy of various combinations is needed.

The soothing and comforting properties of parents’ live lullaby singing are well known [8, 9]. Several systematic reviews have demonstrated the positive effects of music-based interventions for preterm and full-term infants, such as reduced pain levels, improved physiological measures, decreased stress levels, and decreased maternal anxiety [10–12]. When mothers sang and talked to their infants during heel stick, this resulted in increased oxytocin and decreased anxiety in both mothers and infants [13]. Maternal live singing also reduced pain scores during immunizations of two to four-month-old infants [14], and a combination of soothing vocalization and rocking/holding reduced the infants’ crying time during immunizations [15]. However, live parental lullaby singing, informed by music therapy research, has not previously been appraised in a randomized controlled trial (RCT) in combination with SSC and breastfeeding during painful procedures.

Untreated or undertreated pain leaves infants at risk of both short- and long-term consequences. Repeated pain early in life could lead to, for example, altered cortical development [16], altered pain response and increased internalizing behavior [17], long-term effects on cortisol response [18], and alterations in developmental outcomes [19]. Full-term neonates exposed to extreme stress during delivery, or to a surgical procedure, reacted with increased behavioral responsiveness to later painful procedures [20]. Infants, feel, experience, and have a sensory memory of pain, which makes them vulnerable to its negative effects [21, 22]. Neonatal pain research mostly focuses on preterm and sick full-term infants, which is justified, because this population undergoes many painful procedures when staying in neonatal intensive care [23]. However, healthy full-term infants are also routinely exposed to painful procedures, such as vitamin K injections and immunizations, putting them at risk of untreated or undertreated pain early in life.

To our knowledge, this project is the first to explore the efficacy of multi-sensorial parent-delivered pain management combining SSC, breastfeeding, and live parental lullaby singing for full-term infants undergoing routine blood sampling. There is still a knowledge-to–practice gap in neonatal care when it comes to helping parents deliver pain management and a lack of research on combined parent-delivered pain-relieving methods.

## Aim

The aim of this study was to investigate the pain-relieving effects of parent-delivered pain management combining SSC, breastfeeding, and live parental lullaby singing compared with SSC alone and with standard care using oral glucose in healthy newborn infants.

## Methods

### Design

This study was a multicenter RCT with three parallel groups. The groups were assigned at a 1:1:1 allocation. CONSORT guidelines for randomized controlled trials were followed [24] and the study was conducted in compliance with the published protocol [25].

### Participants

Eligibility criteria for the study were healthy newborn infants undergoing routine newborn metabolic screening or other planned venous blood sampling as part of postnatal care. Exclusion criteria were infants treated with sedatives or analgesics within 24 h before the procedure. Parents who understood Swedish or English were eligible for inclusion.

### Study setting

The participants were parent–infant dyads recruited from three healthcare regions in Sweden. Both regional and university hospitals were included. In the Swedish context, the healthy newborn infant is often discharged soon after birth and returns at 48 h of age for a follow-up visit. This was the situation at one of the hospitals, whereas at the other two hospitals, both infants who were still admitted to the maternal unit and those who were discharged and had returned for the 48-hour follow-up visit participated. Participants were enrolled between January 2022 and September 2023.

### Study interventions

The parent–infant dyad was randomized to one of three groups: standard care with oral glucose; skin-to-skin contact; or a combination of SSC, breastfeeding (if applicable), and live parental lullaby singing (Table 1).

**Table 1:** Treatment groups and descriptions of the interventions

Treatment groups	Treatment description
1. Standard care with oral glucose	The infant was placed on the examination table for the blood test and received orally administered glucose according to the standard protocol. Standard care comprised comforting by the parent, oral glucose (300 mg/mL), and the opportunity to suck on a pacifier or the parent’s finger. The parents administered the oral glucose with a syringe after instruction from the health care professional.
2. Parent-delivered pain management with skin-to-skin contact	The parent sat in an adjustable recliner chair during the procedure and the infant was placed naked (except for a diaper and possibly a hat) on the parent’s bare chest 10 min before the venipuncture. The skin-to-skin contact continued during and for a while after the procedure. Infants were allowed to suck on the parent’s finger or a pacifier. Some infants wanted to breastfeed and were allowed to do so for ethical reasons.
3. Parent-delivered pain management combining skin-to-skin contact, breastfeeding, and live parental lullaby singing	In the combined group, the infant was positioned as in group 2, and the infants started breastfeeding at least two minutes before the venipuncture. To ensure intervention fidelity in the live parental lullaby singing, parents were both verbally guided how to sing and shown a short video of a parent singing according to the description of the lullaby singing intervention (the humming should be low-pitched, simple, repetitive, soft, and sedative, with a slow, steady, and predictable pulse in 3/4 or 6/8 time, without sudden shifts or modulations, maintaining a constant recommended sound level of ≤ 55–65 dB) [26]. The parent started humming the lullaby when the infant was placed on the parent’s bare chest 10 min before the venipuncture and continued singing during and for a while after the venipuncture.

### Outcomes

The primary outcome of the study was infant pain expression evaluated using the pain assessment instrument Premature Infant Pain Profile—Revised (PIPP-R) PIPP-R has been validated for procedural pain assessment up until full term [27]. The secondary outcomes were galvanic skin response (GSR), in terms of changes in peaks per second [28], and parents’ ratings, using a visual analogue scale (VAS), of infant pain and of the parents’ own stress and sense of meaningfulness.

PIPP-R is one of the most used scales in research and has been tested for reliability, construct validity, and clinical utility, with the results indicating good psychometrics for both preterm and full-term infants [29]. The scale evaluates three behavioral facial expressions (i.e., brow bulge, eye squeeze, and nasolabial furrow), two physiological measures (i.e., heart rate and oxygen saturation), and two contextual measures (i.e., gestational age and behavioral state). The scores range from 0 to 21, with a higher score indicating an increased level of rated pain. There are three pain score intervals: 1–6 indicates mild pain, 7–12 moderate pain, and 13 and above severe pain. The highest possible score is 18 for full-term infants and 21 for preterm infants [27]. Before the painful procedure, a baseline is assessed by observing the infant for 15 s, measuring the heart rate and oxygen saturation together with observing the infant’s behavioral state and noting the gestational age. Changes from baseline are then assessed for the first 30 s of the painful procedure, starting from when the needle punctured the infant’s skin.

Galvanic skin response (GSR) measures changes in electrical activity following changes in sweat gland activity and is measured using three electrodes placed on the sole of the infant’s foot. An increase in skin conductance reflects the infant’s arousal intensity in response to a painful procedure. Several studies have tested GSR as a measure of neonatal pain for both full-term and preterm infants [28]. In the study protocol [25], we aimed to report all three GSR variables; however, more recent studies report changes in peaks per second to be the most relevant parameter for pain [28, 30, 31], so that is what will be reported here.

For the parents’ ratings, a visual analogue scale (VAS) with a 100-mm line was used. The VAS line was anchored, ranging from “no pain” at the left-hand end point to “worst possible pain” at the right-hand end point. For the other questions, the anchors ranged from “not stressed at all” at the left-hand end point to “worst possible stress” at the right-hand end point, and from “not meaningful at all” at the left-hand end point to “most meaningful” at the right-hand end point of the VAS scale. The parents performed the ratings on the VAS scales directly after the blood sampling procedure ended. The VAS-questionnaire used in the study is included in Appendix A.

### Sample size

The sample size was calculated based on earlier studies using PIPP-R as the outcome and assuming that a difference of two points between groups would be considered clinically important. The standard deviation was also assumed to be two points based on the same studies [33, 34]. A power calculation resulted in 63 infants in each group, with a power of 0.8 and a significance level of 0.05 (www.clinicalc.com). To compensate for incomplete data, for example, from technical issues, blood sample failure, and possible dropouts, 75 infants per group were enrolled for a total sample of 225 infants.

### Randomization

Randomization was conducted using an Internet-based tool (www.random.org) in blocks of 12 per hospital. Documents with the randomization group were printed out and put in sealed opaque envelopes. When the signed consent forms were collected, the envelopes were opened by the researchers and the intervention groups for the parent–infant dyads were revealed.

### Data collection

Demographic data for the parent–infant dyad were collected before the start of the procedure. The parents were also asked about previous music and singing activities during pregnancy.

In all groups, a probe (Philips IntelliVue X3, Eindhoven, NL) was attached to one of the infant’s feet to register heart rate and oxygen saturation, while the electrodes to measure GSR were attached to the other foot. The probe and electrode were attached by the researchers before the procedure and the infant was given time to settle. The infant’s face and pulse oximetry were video recorded for later pain assessment with PIPP-R. The venipuncture was performed by experienced midwifes, nurses, or assistant nurses at each site. When the procedure started (i.e., when the tourniquet was placed), a vocal signal was video recorded and simultaneously noted on the GSR recording; when the needle punctured the skin, a second signal was recorded. The infants were monitored throughout the procedure. If the venipuncture attempt failed, only the first skin puncture was used in the analysis.

### Data analysis

The PIPP-R score was assessed from the video-recordings by MCM, who is a trained neonatal nurse. To assess inter-rater reliability, 20% of the assessments were repeated by EO, who is a nurse experienced in PIPP-R assessment. The GSR values were analyzed using software from MedStorm (Oslo, Norway) by ME, AU, and JE. The parents’ VAS ratings were assessed by AU and EO.

Data were analyzed according to intention to treat (ITT). To analyze whether there were differences between randomization groups for GSR assessments, in which data were normally distributed, an ANOVA with Scheffe post hoc testing was conducted. The ANOVA is presented with means and standard deviations. The PIPP-R and VAS data did not fulfill the criteria for normal distribution; differences between randomization groups were analyzed using the Kruskal–Wallis test and are presented with the medians and interquartile ranges. *P*-values < 0.05 were considered statistically significant. Intra-class correlation (ICC) was used to test the inter-rater reliability of 20% of the PIPP-R scorings, indicating an intra-class correlation of 0.965. Statistical analysis was performed using SPSS version 28 (IBM Corp, Armonk, NY, USA).

## Results

A total of 225 parent–infant dyads participated in the study. The mean birth gestational age of the infants was 39 weeks and four days, and the mean birth weight was 3527 g (see Table 2 for demographic data). Of eligible dyads, a total of 116 parents (34%) declined to participate in the study; their infants did not differ in gestational age or birth weight from the included infants. Because of technical difficulties, 20 (8%) GSR measurements and 13 (6%) PIPP-R assessments were excluded from the analyses.

**Table 2:** Infant characteristics

	Group 1 (*n* = 74)	Group 2 (*n* = 76)	Group 3 (*n* = 75)
Gestational age, weeks, mean (SD)	39.4 (1.4)	39.7 (1.4)	39.8 (1.3)
Birth weight, grams, mean (SD)	3355 (450)	3515 (526)	3531 (546)
Sex, female, *n* (%)	36 (49)	42 (55)	32 (43)
Delivery mode, *n* (%)			
*Vaginal delivery*	66 (89)	71 (93)	65 (87)
*Caesarian section*	4 (5)	3 (4)	8 (11)
*Vacuum extraction*	2 (3)	2 (3)	1 (1)
Time since fed, minutes, median (IQR)	60 (0–90)	0 (0–30)	0 (0–2)
Breastfeeding, *n* (%)	-	18 (24)	65 (87)
Infant sucking at venipuncture, *n* (%)	65 (88)	52 (68)	53 (71)

Pain scores were significantly lower for the standard care group with oral glucose (*p ≤* 0.001). The median PIPP-R scores in all groups were ≤ 7, which is within the mild pain interval and at the lower end of the moderate pain interval (Table 3). There was no significant difference in changes of GSR peaks per second from before to after skin puncture in any of the groups. On the VAS, parents rated their infants’ pain between 9.5 and 17 mm, significantly higher in groups 2 and 3. The parents’ VAS ratings of their own stress were between 4.5 and 6.5 mm (n.s.), and their sense of meaningfulness from participating in their infants’ pain management was rated between 93 and 96 mm (n.s.) (Table 3).

**Table 3:** PIPP-R scores, changes in GSR peaks/sec, and parents’ VAS ratings of infant pain, parents’ own stress, and parents’ sense of meaningfulness

	Group 1 (*n* = 74)	Group 2 (*n* = 76)	Group 3 (*n* = 75)	*p*-value
PIPP-R, median (IQR)	5 (3–6)	7 (5–9)	7 (5–10)	< 0.001
GSR, changes in peaks/sec, mean (SD)	0.002 (0.231)	0.041 (0.213)	0.018 (0.228)	0.587
VAS, infants’ pain, mm, median (IQR)	9.5 (4–21.25)	15.5 (5.25–30)	17 (6–39)	0.017
VAS, parents’ stress, mm, median (IQR)	6.5 (1–33.75)	4.5 (0–20.75)	6 (1–22)	0.344
VAS, parents’ sense of meaningfulness, mm, median (IQR)	94 (73–98)	96 (85.25–100)	93 (81–100)	0.083

No adverse events were observed in any of the groups. The lullabies and songs the parents chose to sing in the combined parent-delivered intervention were mostly Swedish traditional lullabies, whereas a few parents hummed pop songs, songs from movies, and songs reflecting their cultural identity. Some parents sang the same song throughout the procedure and others combined several children’s songs into a medley. The parents’ song choices in treatment group 3 are listed in Appendix B.

## Discussion

To our knowledge, this was the first multicenter RCT to evaluate combined parent-delivered pain management including parents’ live singing compared with SSC and standard care. Pain scores were low in all groups (within the mild pain interval and at the lower end of the moderate pain interval), but significantly lower for the standard care group with oral glucose. No significant differences were found in peaks per second measured in terms of GSR. The VAS score results indicated low stress and considerable meaningfulness for the parents, who were all participating in their infants’ pain management in various ways.

All infants have the right to effective pain management during painful procedures. Most (70%) international neonatal clinical pain trials that study pain-relieving interventions include a no-treatment or placebo control group in their studies, meaning that these studies purposely expose infants to unnecessary harm [32]. In this study, we took all the necessary precautions to ensure the ethical conduct by providing evidence-based pain management for all included infants. It was hypothesized that the combined parent-delivered pain management would be more effective in alleviating the infants’ pain than skin-to-skin contact alone or standard care with oral glucose. However, the primary hypothesis was not supported. There is a lack of previous studies comparing SSC or combined parent-delivered pain management to oral glucose or sucrose in healthy full-term infants, and several studies have been performed with no treatment as control. Future research should be done to include sweet solutions in the combined pain management.

Sweet oral solutions such as sucrose and glucose are part of standard care in neonatal pain care globally. Sweet solutions are considered a pharmacological agent and have been extensively investigated and found to reduce procedural pain from blood-sampling procedures in both preterm and full-term infants without serious side effects [33, 34]. Because it is recommended that pharmacological pain therapies should be used in conjunction with non-pharmacological interventions [35], and because non-pharmacological interventions should in general be the first choice in procedural pain management [36], sweet solutions might, for example, be more effective in combination with non-pharmacological interventions than sucrose or glucose alone, which some studies show [34, 37], but more research is needed to confirm this. Including the parents’ love, comfort, and support in a painful context would facilitate participation, closeness, and parenting capacity as well as decreasing stress in the dyad, even for parents with needle phobia [1]. For healthcare, this is also an investment addressing future painful procedures.

Pain assessment in non-verbal children and infants is challenging and there is no consensus on how to best evaluate their pain. In addition, there is no previous research in full-term infants guiding the pain assessment and pain score analysis of combined multisensorial and multimodal parent-delivered methods like the one administered in group 3 in this RCT, which also included the parents’ interactive live singing. The three parent-delivered interventions together stimulated the infants’ tactile, gustatory, auditory, visual, olfactory, and vestibular senses, theorizing that this bundle of sensorial input would activate the descending pain modulatory system and inhibit the nociceptive signals in the newborn [38]. Unlike Nimbalkar’s [38] study, our study did not establish that SSC and sweet solutions have comparable efficacy in reducing pain. Further research is needed to adequately capture and assess combined multisensorial pain management.

When assessing pain, the best approach is a multimodal model combining behavioral pain scales with various biomarkers, for example, GSR [39]. In this study, no significant relationships were found between the behavioral pain scores and changes in GSR. Discrepancies between behavioral assessments and skin conductance have been identified in other studies as well, which found no significant relationships between pain assessment scales and skin conductance [28]. Absence of a physiological indication of pain does not necessarily mean that the infant is free from pain. Perhaps the choice of another biopsychological measure could have been useful for this study [22, 40]. The PIPP-R also involves physiological measures of pain, such as heart rate and oxygen saturation, which also add a physiological component to the measurements. The nonalignment of the results of the primary and secondary outcomes might have been related to the potential arousal in the infants in reaction to handling. The power calculation for this study was made with PIPP-R as the main outcome, not taking the biopsychological aspect into consideration. The infants in this second part of SWEpap were healthy full-term infants, whereas much previous research on parent-delivered pain management has been performed on preterm infants. There are indications that brain maturation could explain why full-term infants better communicate their pain experience through facial expressions [41]. The PIPP-R assessment should however have been able to recognize the infants’ pain reactions properly due to the facial parameters included in the scale.

The parents in this study rated their infants’ pain as low in all groups, particularly in the oral glucose group. The parents’ VAS scoring regarding their infants’ pain corresponded well with the PIPP-R intervals from the assessment by the trained nurses and authors of this study, although there were slightly higher points in the PIPP-R assessment than in the parents’ VAS ratings. This shows the parents’ ability to accurately assess their infants’ pain. In other studies, the pain assessments have differed between parents and healthcare professionals, with parents rating the pain both higher and lower than did the nurses, suggesting that parents may have been influenced by other aspects than only the infants’ pain expressions [42]. Parents’ ability to assess pain could be a useful and important resource in the collaborative partnership between healthcare professionals and parents, and it may also empower parents when intuitively comforting their infants during painful procedures. Parents need information, preparation, and support to assess their infants’ pain [1].

This is the first study to include parents’ live lullaby singing in combined parent-delivered pain management during painful procedures. Music therapy and parents’ voices can play a crucial part in infants’ pain relief, as previously confirmed in systematic reviews [12] and in the qualitative part of the SWEpap project [1]. The parents’ live singing was considered an accessible resource for both parents and infants during painful procedures, enhancing the parents’ emotional availability and responsiveness in the situation, helping the parents and infants calm down before the procedure, and creating a calm and trusting atmosphere during the procedure for everyone, including the healthcare professionals [1]. The SWEpap study, parts 1 and 2, has investigated this unexplored relational and interactive intervention, informed by music therapy, as a pain-alleviating adjuvant in combination with SSC and breastfeeding. As indicated by the parents’ VAS ratings in group 3, and in line with previous research showing decreased stress and anxiety in parents who interact vocally with their infant [13], this intervention seems to be a feasible supplement that could be useful even in the stressful outpatient clinic.

In this study, all parents were actively involved in their infants’ pain management and said that they were positive about being involved, assigning a high meaningfulness score in all treatment groups. This is in line with what we know from previous research regarding parents’ readiness and need to be active in their infants’ pain management [2]. As for participating in pain research, parents have reported feelings of being useful, reassured, calm, and empowered after participating in their infants’ pain management in a study setting [43]. Parents are increasingly involved in their infants’ care since the concept of family-centered care (FCC) has been integrated into neonatal care, especially in the Nordic countries [44]. Family-centered care principles emphasize parental presence, participation in the care of the infant, and shared decision making [45]. Still, we know that parents are not always invited to participate in their infants’ pain management. Healthcare professionals should take an active role in inviting, informing, and preparing parents to be active in delivering their infants’ pain management. In addition, proactive information and directed knowledge transfer to expecting parents could increase the use of parent-delivered pain management strategies and parents’ confidence in their ability to manage and assess infant pain [46].

### Strengths and limitations

Before testing this unexplored concept for parent-delivered pain management combining SSC, breastfeeding, and parents’ live lullaby singing in a larger sample, the feasibility of the design was first qualitatively explored in the neonatal intensive care context with premature infants [1]. The 10-minute preparation time for the combined parent-delivered pain management was found feasible in this first part of the SWEpap project [1]. The differing contexts of SWEpap parts 1 and 2 studies are a limitation, with the extra preparation time needed for the parent–infant dyads considered feasible in the neonatal care setting perhaps being considered overly time consuming in outpatient care by staff subject to time constraints. The lack of time could perhaps also be a reason why some parents declined to participate in SWEpap 2.

There was initially no previous research on a parent-delivered pain intervention combining SSC, breastfeeding, and live parental singing to guide us in the optimal duration of the combination for the method to be effective. To ensure the analgesic effect of breastfeeding, the infant must be latched and sucking well at least two minutes before the blood test is performed [47]. For an analgesic effect with SSC, previous research has stated various options ranging from 10 to 30 min before the procedure [5, 48]. The optimal duration for achieving the analgesic effect of music-based interventions, live or recorded, is still unknown [12]. However, based on evidence collected over the last decade, it is recommended that parents’ live singing with preterm infants should start up to five minutes before a blood sampling procedure, continue throughout the procedure and for five minutes afterward to decrease infants’ pain levels, increase oxytocin levels in both infants and parents, and decrease the parents’ anxiety [9, 49].

The challenge of a protocol-based design was identified by Hauck et al. [50] in an RCT intended to assess whether parental stroking of their infant before or after a heel lance could provide effective pain relief. The trial emphasized the challenge of translating an experimental researcher-led tactile intervention into a parent-led approach, with parents following a protocol rather than their own sense of how to stroke their infant. If parents could intuitively decide how to comfort their infant and for how long before the skin puncture, the intervention could be tailored to what is best for the unique parent–infant dyad, and perhaps the effect could be improved. Future studies should aim to involve parents in these decisions.

No blinding was possible because it was obvious in the recordings whether the infants were placed skin-to-skin, breastfed, or placed on the examination table. Sound was also recorded with verbal notifications to facilitate the pain assessment. This could entail bias on the part of the person performing the pain assessment. However, the high inter-rater reliability increases the strength of the pain assessments despite the lack of blinding. Including a large number of full-term infants from multiple centers, together with the lack of differences between the participating infants and the infants whose parents declined to participate, strengthens the external validity of the study.

The use of VAS could be a limitation, since VAS is a subjective measure and relies on the parent’s interpretation of the child’s behavior and signals. There is limited evidence that the VAS applied by a parent is reliable for assessing pain in infants [51]. Regarding VAS-assessments for stress and meaningfulness there is no earlier studies investigating the psychometrics. However, the VAS is a commonly used tool for self-assessment in different settings and could be convenient also in this setting.

Another strength of our study is that parent–infant dyads with various cultural backgrounds were included; this could increase the generalizability of the study, although the participants’ cultural backgrounds are not reported for ethical reasons. Both mothers and fathers participated in the study, as well.

## Conclusions

Pain scores in this RCT remained within the mild to moderate range across all groups, with the infants receiving oral glucose having significantly lower pain scores. With a mixed-methods design, the SWEpap project was the first to investigate both the feasibility and efficacy of parent-delivered pain management combining SSC, breastfeeding, and live parental lullaby singing during routine blood sampling in preterm and full-term infants. More research in parent-delivered neonatal pain management is needed to optimize, for example, the measurement methods and the durations of the various steps of the experimental method. Parent-delivered pain management combining SSC, breastfeeding, and parents’ live lullaby singing is a feasible and safe intervention with potential pain alleviating properties offering the parents a strong sense of meaningfulness and stress relief.

## Supplementary Information

Supplementary Material 1.

Supplementary Material 2.

Supplementary Material 3.

Supplementary Material 4.

Supplementary Material 5.

## Data Availability

The datasets used and/or analyzed in this study are available from the corresponding author on reasonable request.

## Abbreviations

GSR: Galvanic skin response
SSC: Skin-to-skin contact
PIPP-R: Premature Infant Pain Profile—Revised
RCT: Randomized controlled trial
VAS: Visual analogue scale
