# Supplementary material for: Effect of combined skin-to-skin contact, breastfeeding, and parents’ live lullaby singing on relieving acute procedural pain in neonates (SWEpap): a multicenter randomized controlled trial in Sweden
Source: BMC Pediatr. 2025 Dec 10;26:37. doi: 10.1186/s12887-025-06393-y (PMC12817788; doi:10.1186/s12887-025-06393-y)
Supplement: Supplementary file 1 — Supplementary Material 1. [file 12887_2025_6393_MOESM1_ESM.doc]

| **Study period** | | | | | | | |
| --- | --- | --- | --- | --- | --- | --- | --- |
|  | **Enrollment** | | **Allocation** | **Post allocation** | | | |
| **TIMEPOINT**** | ***-t_1_***  Before discharge from the unit | **-t_2_**  Upon arrival at the unit | **0** | **t_1_**  Baseline | **t_2_**  Venipuncture | **t_3_**  Post venipuncture | **t_4_**  After procedure |
| **Enrollment:** |  |  |  |  |  |  |  |
| Eligibility screening | X |  |  |  |  |  |  |
| Written information | X |  |  |  |  |  |  |
| Informed consent |  | X |  |  |  |  |  |
| **Allocation** |  |  | X |  |  |  |  |
| **Interventions:** |  |  |  |  |  |  |  |
| Standard care |  |  |  |  |  |  |  |
| SSC |  |  |  |  |  |  |  |
| SSC+ BF |  |  |  |  |  |  |  |
| SSC + BF + PIDS |  |  |  |  |  |  |  |
| **Assessments:** |  |  |  |  |  |  |  |
| Demographic data |  |  | X |  |  |  |  |
| Heart rate and oxygen saturation probe connected |  |  | X |  |  |  |  |
| GSR connected |  |  | X |  |  |  |  |
| Video cameras in position |  |  | X |  |  |  |  |
| **Outcome variables** |  |  |  |  |  |  |  |
| PIPP-R |  |  |  | X | X | X |  |
| GSR |  |  |  | X | X | X |  |
| VAS-rating for parents |  |  |  |  |  |  | X |
| **Abbreviations:** SSC = Skin to skin contact BF= Breastfeeding PIDS= Live parental infant-directed lullaby singing | | | | | | | |

Figure 1. Timeline of the study
