# Supplementary material for: Effect of combined skin-to-skin contact, breastfeeding, and parents’ live lullaby singing on relieving acute procedural pain in neonates (SWEpap): a multicenter randomized controlled trial in Sweden
Source: BMC Pediatr. 2025 Dec 10;26:37. doi: 10.1186/s12887-025-06393-y (PMC12817788; doi:10.1186/s12887-025-06393-y)
Supplement: Supplementary file 2 — Supplementary Material 2. [file 12887_2025_6393_MOESM2_ESM.doc]

**CONSORT 2010 Flow Diagram**

**Allocation**

**Analysis**

**Enrollment**

Assessed for eligibility (n=341)

Excluded (n=116)

♦  Declined to participate (n=116 )

Analyzed (n=74)
♦ Excluded from PIPP-R analysis (missing data) (n=6)

♦ Excluded from GSR analysis (missing data) (n=5)

Allocated to intervention (n=74)

♦ Received allocated intervention (n=74)

♦ Did not receive allocated intervention (give reasons) (n=0)

Allocated to intervention (n=75)

♦ Received allocated intervention (n=75)

♦ Did not receive allocated intervention (give reasons) (n=0 )

Analyzed (n=75)
♦ Excluded from PIPP-R analysis (missing data) (n= 4)

♦ Excluded from GSR analysis (missing data) (n=6)

Randomized (n= 225)

Allocated to intervention (n=76)

♦ Received allocated intervention (n=76)

♦ Did not receive allocated intervention (give reasons) (n=0)

Analyzed (n=76)
♦ Excluded from PIPP-R analysis (missing data) (n= 3)

♦ Excluded from GSR analysis (missing data) (n=4)
