# Supplementary material for: Effect of combined skin-to-skin contact, breastfeeding, and parents’ live lullaby singing on relieving acute procedural pain in neonates (SWEpap): a multicenter randomized controlled trial in Sweden
Source: BMC Pediatr. 2025 Dec 10;26:37. doi: 10.1186/s12887-025-06393-y (PMC12817788; doi:10.1186/s12887-025-06393-y)
Supplement: Supplementary file 4 — Supplementary Material 4. [file 12887_2025_6393_MOESM4_ESM.docx]

***Appendix B. Parents’ song choices in group 3***

Alla ska sova för nu är det natt (Lindgren A/Riedel G, 1986, Sweden)

Auld lang syne (Burns R/Shield W, 1788, UK)

Babblarnas vaggvisa (Tisell A / Sjölander H / Rask J, 2017, Sweden)

Baby Beluga (Raffi, 1979, Canada)

Bajka iskierki (Trad., Porazińska J, 1925, Poland)

Björnen sover (Trad., Sweden)

Blinka lilla stjärna (Trad., Twinkle, twinkle, little star)

Byssan lull (Taube E, 1919, Sweden)

Bä bä vita lamm (Tegnér A, 1892, Sweden. Translated from Baa baa black sheep, 1744)

Det vackraste (Grönvall P/Rådsten M/Grönvall N,1995, Sweden)

Ek from Majas alfabetssånger (Andeby K, 1995, Sweden)

En liten båt (Hellsing L, 1994, Sweden)

Idas Sommarvisa (Lindgren A/Riedel G, 1973, Sweden)

I ett hus vid skogens slut (Trad., Sweden)

Imse vimse spindel (Trad., Itsy Bitsy Spider)

Kattvisan (Lindgren A/ Söderlundh LB, 1955, Sweden)

Krokodilen i bilen (Forsberg M/Andeby K,1998, Sweden)

Kurdish lullaby (not specified)

La la lu (Lee P/Burke S, 1955, USA )

Lilla snigel (Trad., 1957, Sweden)

Lille katt (Lindgren A/Riedel G, 1971, Sweden)

Må solen alltid skina (Palsdottir E, 2009, Faroe Islands)

Nu i ro slumra in (Wiegenlied, Brahms J, 1868, Germany)

Smedsvisa (Trad., folksong, Sweden)

Sjörövar-Fabbe (Lindgren A/Riedel G, 1970, Sweden)

Somali lullaby (not specified)

Sommarpsalm (af Wirsén C D/ Gastorius S, 1889, Sweden)

Sommartider (Gessle P, 1982, Sweden)

Trollmors vaggsång (Holmberg M, 1943, Sweden)

Tula hem och tula vall (Tegnér A, 1892, Sweden)

Vaggvisa för flyktbenägna (Hellström H, 2005, Sweden)

Vargsången (Lindgren A/Isfält B, 1984, Sweden)

Vem kan segla förutan vind (Samuelsson L, 1971, Åland )

Vyssa, lulla litet barn (Trad., 1842, Sweden)

You´ll be in my heart (Collins P, 1999, UK)
