# Supplementary material for: Effect of combined skin-to-skin contact, breastfeeding, and parents’ live lullaby singing on relieving acute procedural pain in neonates (SWEpap): a multicenter randomized controlled trial in Sweden
Source: BMC Pediatr. 2025 Dec 10;26:37. doi: 10.1186/s12887-025-06393-y (PMC12817788; doi:10.1186/s12887-025-06393-y)
Supplement: Supplementary file 5 — Supplementary Material 5. [file 12887_2025_6393_MOESM5_ESM.docx]

## **Appendix A. VAS-questionnaire used in the study**

1. **How do you assess your infant’s pain during the blood sampling?**

**(mark with an X on the line**)

No pain Worst possible pain

1. **How stressed did you as a parent feel during the blood sampling?**

Not stressed at all Worst possible stress

1. **For you personally, how meaningful was it to take part in the pain management during your infant’s blood sampling?**

Not meaningful at all Most meaningful

**Comments:**
